# Activation of N-methyl-D-aspartate receptor regulates insulin sensitivity and lipid metabolism

**DOI:** 10.7150/thno.51666

**Published:** 2021-01-01

**Authors:** Xiao-Ting Huang, Jun-Xiao Yang, Zun Wang, Chen-Yu Zhang, Zi-Qiang Luo, Wei Liu, Si-Yuan Tang

**Affiliations:** 1 Xiangya Nursing School, Central South University, Changsha, Hunan, China; 2 Department of Orthopedics, Xiangya Hospital, Central South University, Changsha, Hunan, China; 3 Department of Physiology, School of Basic Medicine Science, Central South University, Changsha, Hunan, China

**Keywords:** Obesity, Insulin resistance, Nonalcoholic fatty liver disease, Glutamate, N-methyl-D-aspartate receptor, PPARα

## Abstract

Rationale: Although significant progress has been made in understanding the mechanisms of steatosis and insulin resistance, the physiological functions of regulators in these processes remain largely elusive. Evidence has suggested that the glutamate/N-methyl-D-aspartic acid receptor (NMDAR) axis contributes to acute lung injury, pulmonary arterial hypertension, and diabetes, but the specific metabolic contribution of the glutamate/NMDAR axis is not clear. Here we provide data at the animal, cellular, and molecular levels to support the role of the glutamate/NMDAR axis as a therapeutic target for metabolic syndrome in obesity.

**Methods:** We examined the glutamate level in the obese mouse induced by a high-fat diet (HFD) for 12 weeks. To assess the role of NMDAR in insulin sensitivity and lipid metabolism, we tested the effects of Memantine (an NMDAR antagonist) and NMDA (an NMDAR agonist) on mice fed with HFD or standard chow diet. The *in vitro*s NMDAR roles were analyzed in hepatocytes and potential mechanisms involved in regulating lipid metabolism were investigated.

**Results:** Glutamate was increased in the serum of HFD-treated mice. The NMDAR blockade by Memantine decreased the susceptibility to insulin resistance and hepatic steatosis in obese mice. NMDA treatment for 6 months induced obesity in mice, characterized by hyperglycemia, hyperlipidemia, insulin resistance, and pathological changes in the liver. We provided *in vitro* evidence demonstrating that NMDAR activation facilitated metabolic syndrome in obesity through promoting lipid accumulation. NMDAR inhibition attenuated lipid accumulation induced by palmitic acid. Mechanistically, NMDAR activation impaired fatty acid oxidation by reducing PPARα phosphorylation and activity. The PPARα activity reduction induced by NMDAR activation was reversibly mediated by ERK1/2 signaling.

**Conclusion:** These findings revealed that targeting NMDAR might be a promising therapeutic strategy for metabolic syndrome in obesity.

## Introduction

Obesity is caused by excessive accumulation and/or abnormal distribution of fat that presents a health risk 1. The prevalence of obesity has expanded dramatically all around the globe, contributing to an increased risk for metabolic syndrome, nonalcoholic fatty liver disease (NAFLD), type 2 diabetes (T2D), cancer, and heart disease 2-6. These associated complications increase the health burden in many countries 7. Food and Drug Administration (FDA) has approved drug therapies for obesity, including orlistat, phentermine/topiramate, lorcaserin, combination naltrexone/bupropion, and liraglutide 8. These drugs improve the quality of life and are used for long-term weight management in adults with a body mass index (BMI) of ≥30 kg/m^2^ or BMI of ≥27 kg/m^2^ in the presence of weight-related comorbidities such as T2D or hypertension. However, the high price and side effects cannot be ignored 9, emphasizing that the need for more effective therapies.

In obese subjects, excess lipids are shunted to non-adipose tissues, such as the liver, heart, and pancreas 1,10. The ectopic fat deposition interferes with insulin signaling and causes insulin resistance 11,12. It is also widely accepted that ectopic lipid accumulation can lead to hepatic steatosis 13,14. Although ectopic lipid accumulation's pathogenesis is not well understood, lipid metabolism in the liver is dysregulated when the balance between fatty acid synthesis and fatty acid oxidation (FAO) is chronically disrupted 15-17. FAO is the mitochondrial aerobic process of breaking down a fatty acid into acetyl-CoA units 16,18. Hepatic FAO disorder is thought to be a crucial process in lipid accumulation and hepatic steatosis 13,19. However, the underlying mechanisms for hepatic FAO disorder are mostly unclear.

Glutamate, an important excitatory neurotransmitter, can be released from tissues other than the brain 20. We and others have reported that endogenous glutamate contributes to T2D, acute lung injury, and pulmonary arterial hypertension 21-24. The increased release of glutamate has also been demonstrated in obesity 25-27, indicating an excessive glutamate increase may be involved in lipid accumulation. Glutamate causes perivenular hepatocellular ballooning, inflammation, and mild hepatic fibrosis and is, therefore, studied in animal models for obesity and steatosis 28-31. Other studies showed that glutamate causes hepatic injury by ablating cells in the hypothalamus' arcuate nucleus and destroying circumventricular neurons 32. Some other studies suggested that glutamate has deleterious effects on the liver at higher doses, a non-neural effect of glutamate 33,34. However, the exact mechanism of glutamate-induced obesity and steatosis, except for ablating circumventricular neurons, is still unknown. Glutamatergic communication through N-methyl-d-aspartate receptors (NMDAR) occurs in both the central nervous system (CNS) and outside the CNS 23,24,35-37. Glutamate contributes to T2D, acute lung injury, and pulmonary arterial hypertension through excessive activation of NMDAR 21-24. Correspondingly, blocking NMDAR with specific antagonists (Memantine (Mem), MK-801, or dextromethorphan) may benefit these conditions 22-24,36. These data strongly suggest that the excessive increase of glutamate contributes to obesity and steatosis through the activation of NMDAR. Therefore, we set out to investigate the glutamate/NMDAR axis role in insulin sensitivity and lipid metabolism.

In this study, we investigated whether blockade of NMDAR by Mem could alleviate obesity-induced insulin resistance and lipid accumulation and explored potential underlying mechanisms.

## Methods

### Animals

The Ethics Committee of the Center for Scientific Research with Animal Models at Central South University (Changsha, China) approved the experiments performed under the National Institutes of Health guidelines. Mice were anesthetized, and necessary efforts were taken to minimize suffering before performing operations.

### Treatment protocols

Eight-week-old male C57BL/6J mice (*n* = 40 per group) were used in this study. All experiments were conducted using non-littermate male mice. After a one-week adaptation period, mice were fed with a high-fat diet (HFD, 506 kcal/100 g, 60.0% energy as fat) or a standard chow diet (359 kcal/100 g, 12.8% energy as fat). Mem (5 mg/kg) or saline was intraperitoneally injected daily from week 1 to week 12 to examine if blockade of NMDAR with Mem could prevent obesity phenotypes. To investigate whether NMDAR blockade by Mem could ameliorate obesity phenotypes, Mem (5 mg/kg) or saline was intraperitoneally injected daily from week 13 to week 24. The role of NMDAR activation in lipid accumulation and glucose homeostasis of mice fed with standard chow diet was analyzed by daily intraperitoneal injection of NMDA (8 mg/kg, Sigma-Aldrich, USA) or saline from month 1 to month 6.

### Food intake studies

Food intake was measured daily for one week. During this period, mice were housed in individual cages. Caloric intake and food intake were estimated using the following formulas: food intake/body weight (g/g) = (initial weight of food provided - final weight of food recovered)/body weight. Food intake/body weight (Kcal/g) = (initial weight of food provided - final weight of food recovered) × Kcal per g diet/body weight. The food consumption was measured daily at the same time (9.00-10.00 am) 38,39.

### Intraperitoneal glucose tolerance test (IGTT) and intraperitoneal insulin tolerance test (IITT)

For the IGTT, mice were fasted for 12 h and then received an intraperitoneal injection of glucose (2 g/kg body weight). Blood glucose concentrations were measured at 0, 30, 60, and 120 min after the glucose injection. For the IITT, mice were fasted for 4 h and intraperitoneally injected with insulin (1 U/kg body weight). Blood glucose concentrations were measured at 0, 30, 60, and 120 min after the insulin injection.

### Blood parameters

The concentrations of the hepatic enzymes alanine aminotransferase (ALT), aspartate aminotransferase (AST), and alkaline phosphatase (ALP) in mice sera were measured by a spectrophotometer (Chemix 180i) according to manufacturer's instructions. Insulin levels in plasma were determined by a mouse insulin ELISA kit (Millipore). The IL-6 and TNF-α levels in the serum were measured by ELISA kits (BD Bioscience). The serum was collected for glutamate measurement using a glutamate detection kit (Nanjing Jiancheng Bioengineering, Nanjing, China).

### Lipid measurement

Serum total cholesterol (TCHO) and low-density lipoprotein (LDL) measurements were measured by using commercial assay kits (Nanjing Jiancheng Bioengineering, Nanjing, China).

### RNA isolation and Real-time PCR (RT-PCR)

Total RNA was isolated from tissue and cells in TRIzol (Thermo Fisher, Waltham, MA). Complementary DNA (cDNA) was prepared by reverse transcribing 1 μg of RNA with a cDNA synthesis kit (Bio-Rad, Hercules, CA). The primers used in the study are listed in Table S1. RT-PCR was performed using SYBR Green Mix (Takara Bio Inc.) on a CFX96 Touch RT-PCR Detection System (Bio-Rad). The comparative Ct (2^-ΔΔCt^) method was used to determine the relative mRNA expression, normalized against β-actin. Fold-changes and statistical significance were calculated from three independent replicates 40.

### Histology and immunohistochemistry

Subcutaneous (SubQ) fat and liver tissues were fixed in 10% neutral-buffered formalin for 24 h and sectioned at 4-μm. H&E staining was performed according to the standard protocols. For Oil Red O staining, liver tissue was fixed in 1% formalin for 2 h and embedded in OCT compound. The tissue was sectioned at 10-µm and stained with Oil red O according to standard methods. For immunohistochemical analysis, pancreatic tissue was fixed in 1% formalin for 2 h and embedded in OCT compound. Sections were treated with a blocking agent (0.5% bovine serum albumin, Sigma-Aldrich, USA) for 45 min at room temperature. Sections were incubated overnight at 4 °C with rabbit anti-insulin antibody (1:50, Abcam, USA) and mouse anti-NMDAR1 antibody (1:50, Abcam, USA). Subsequently, sections were incubated with FITC-conjugated secondary antibody and were observed under a fluorescence microscope (Thermo, USA). The score of liver steatosis was according to the grade of the lesion, slight (0.5), mild (1), moderate (2), severe (3), profound severe (4), and normal (0).

### Cell culture

AML-12 mouse hepatocytes were cultured as instructed. Cells were cultured at 37 °C with 5% CO_2_ in a humidified atmosphere. After 24 h serum starvation, cells were exposed to palmitic acid (200 μM, Sigma-Aldrich, USA). For Oil Red O staining, AML-12 cells were cultured in 24-well plates and incubated with palmitic acid (200 μM) for 24 h. AML-12 cells were cultured with PPARα agonist WY14643 (5 μM, MedChemExpress, USA) 30 min before NMDA administration. Then the cells were collected 24 h after the NMDA administration. HepG2 cells were maintained in Dulbecco's Modified Eagle's Medium (HyClone, Thermo Scientific, USA) supplemented with 10% fetal bovine serum (FBS, Gibco, Rockville, USA) containing 100 U/mL penicillin and 100 μg/mL streptomycin. HepG2 cells were cultured in 24-well plates and incubated with NMDA (5 mM) for 24 h.

### Luciferase assay

AML-12 cells were transfected with the luciferase-reporter plasmid PPRE-Luc [containing three copies of PPRE (PPARα response element) consensus sequence] or the control plasmid. Twenty-four hours later, cells received PPARα agonist WY14643 (5 μM) with or without NMDA (5 mM) for another 24 h. Luciferase activity was determined with a luciferase reporter assay system (Promega, USA). Results were averaged over three biological replicates.

### Western blot analyses

Hepatic tissues were dissected and immediately frozen in liquid N_2_. AML-12 cells extracts were prepared in ice-cold buffer (1% Triton X-100, 50 mM HEPES, pH 7.5, 150 mM NaCl, 1 mM EDTA, 10% glycerin, 10 mM Na_4_P_2_O_7_, 20 mM glycerophosphate, 10 mM NaF, 10 mM sodium orthovanadate and proteinase inhibitor mixture) until the cells were completely lysed. The protein concentrations were measured using a bicinchoninic acid protein assay kit (Sigma-Aldrich, USA). Total protein (40 μg) was resolved on 8%-12% SDS-PAGE gels and transferred onto polyvinylidene difluoride membranes (Millipore, USA). Blotted membranes were then incubated by either anti-ERK (1:1000, CST), anti-phospho-ERK (1:2000, CST), anti-PPARα (1:200, Abcam), anti-phosphoPPARα (Ser12) (1:1000, Abcam), anti-Collagen I (1:1000, Abcam), anti-collagen III (1:2000, Abcam), or β-tubulin (1:1000, CST) antibodies. After several washes, the membranes were incubated with horseradish peroxidase-conjugated anti-rabbit IgG (1:5000, Sigma-Aldrich) or anti-goat IgG (1:5000, Signalway Antibody).

### Statistical analysis

Animals used in experiments of this study were randomly grouped. Histology was performed and analyzed in a double blinded way. Data are expressed as the mean ± SEM. Statistical analysis was performed with SPSS19.0. Unpaired Student's *t*-test (two groups) and ANOVA (multiple groups) were used. Differences were considered significant when *P*-value < 0.05.

## Results

### Blockade of NMDAR by Mem prevents obesity phenotypes on HF diet

**Figure 1:**
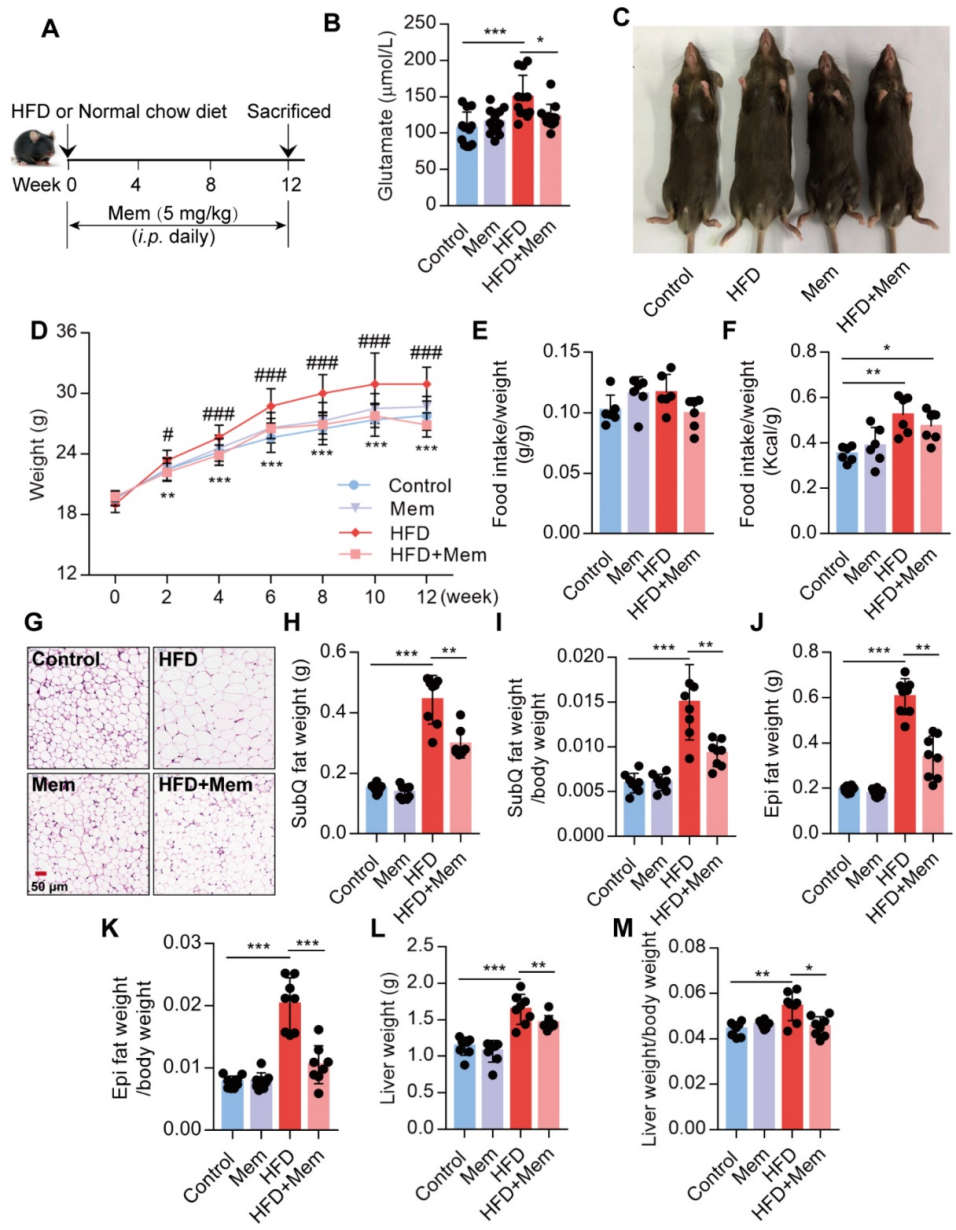
** Blockade of NMDAR by Mem prevents HFD-induced obesity phenotypes in mice.** (A) C57BL/6 mice received intraperitoneal injections of Mem from week 1 to week 12 with HFD or normal chow diet. (B) Glutamate concentration in serum from mice fed with HFD for 12 weeks (*n* = 12). (C) Representative images of mice after HFD feeding for 12 weeks. (D) Growth curve of mice fed with HFD or normal chow diet for 12 weeks (*n* = 12). Compare to the Control group, **P < 0.01; ***P <0.001. Compare to the HFD group, #*P* < 0.05; ###*P* <0.001. (E-F) Determination of food intake of mice fed with HFD or normal chow diet for 12 weeks (*n* = 6). (G) H&E staining of SubQ fat tissue from mice fed with HFD or normal chow diet for 12 weeks (Scale bars = 50 μm). (H-M) Determination of SubQ and Epi fat weight and liver weight from mice fed with HFD or normal chow diet for 12 weeks (*n* = 8). *P < 0.05; **P <0.01; ***P <0.001. All data are presented as the mean ± SEM.

It has been reported that the glutamate level is significantly increased in the serum of obese patients 25,26. Since NMDAR primarily meditate glutamate neurotoxicity, we placed mice with/without Mem (NMDAR antagonist) on a normal chow diet or HFD for 12 weeks (Figure 1A). Consistent with earlier reports, prolonged HFD consumption results in an increased glutamate level in the serum, which was decreased by Mem administration (Figure 1B, Table 1). We found a noticeable increase in the total body weight of HFD-fed mice compared to the control mice, while HFD-fed mice receiving Mem showed significantly lower body weights than HFD-fed mice (Figure 1C-D). There was no difference in the ratio of food intake to body weight (Figure 1E). The calorie intake of HFD-fed mice was higher than that of standard chow diet-fed mice but had no statistical difference with or without Mem treatment (Figure 1F, Figure S1A). At the 12-week time point, H&E staining of SubQ adipose tissues revealed significantly decreased adipocyte diameter in mice of the HFD + Mem group compared with the HFD group (Figure 1G). Furthermore, both SubQ and Epi fat weights were lower in Mem-treated HFD mice than HFD mice (Figure 1H-K). Besides, Mem-treated HFD mice showed a minor increase in liver weight and liver weight ratio to body weight (Figure 1L-M). These data indicated resistance to the HFD-induced obesity phenotype in Mem-treated mice.

### Blockade of NMDAR by Mem prevents insulin resistance and promotes glucose tolerance on HF diet

**Figure 2:**
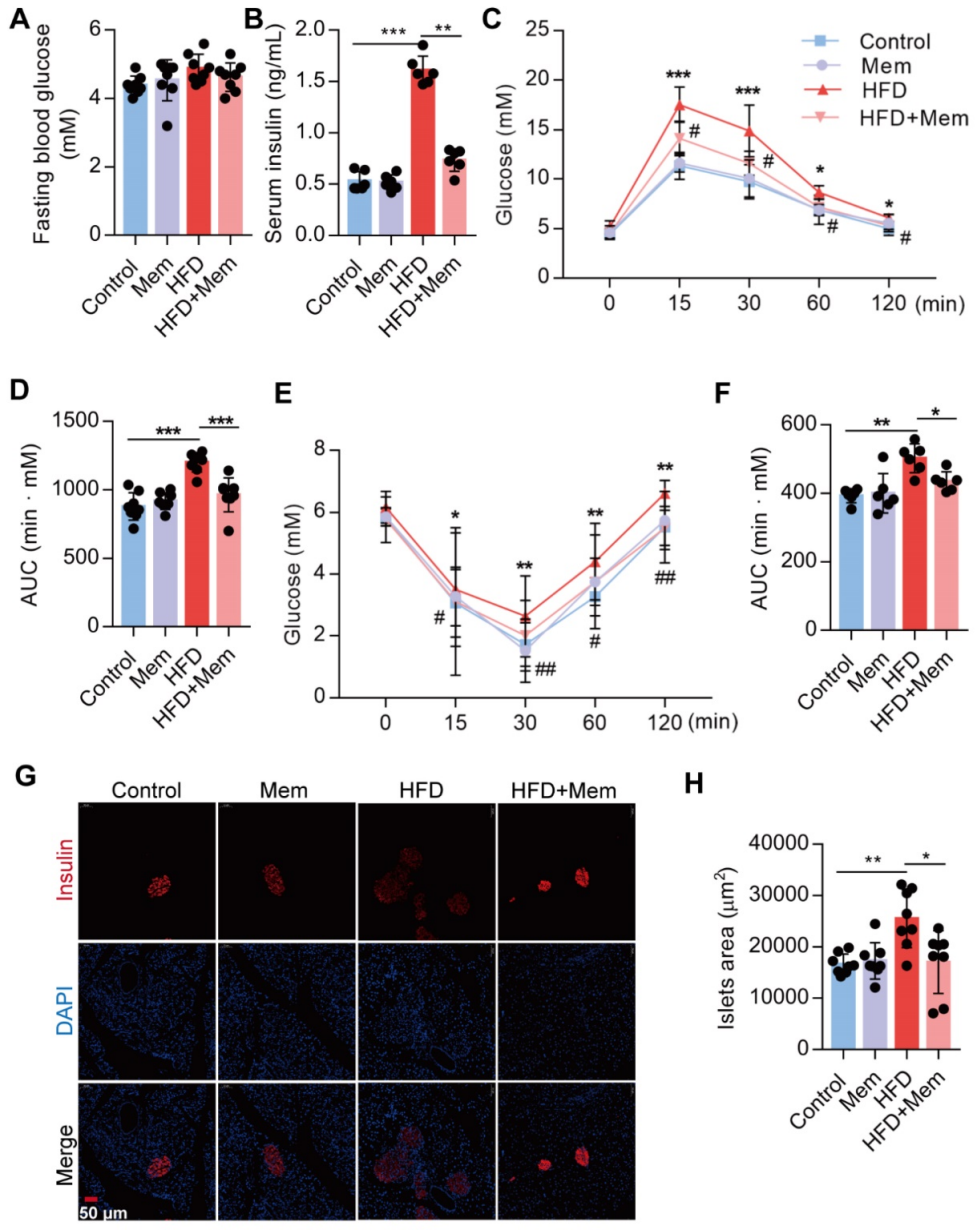
** Blockade of NMDAR by Mem attenuates insulin resistance glucose tolerance of mice fed with HFD.** (A) Measurement of fasting blood glucose of mice fed with HFD or normal chow diet for 12 weeks (*n* = 8). (B) Measurement of serum insulin level of mice fed with HFD or normal chow diet for 12 weeks (*n* = 6). (C-D) Measurement of blood glucose during IGTT of mice fed with HFD or normal chow diet for 12 weeks (*n* = 8). Compare to the Control group, *P < 0.05; ***P <0.001. Compare to the HFD group, #*P* < 0.05. (E-F) Measurement of blood glucose during the IITT of mice fed with HFD or normal chow diet for 12 weeks (*n* = 6). Compare to the Control group, *P < 0.05; **P <0.01. Compare to the HFD group, #*P* < 0.05; ##*P* <0.01. (G-H) Representative immunofluorescence staining images of pancreases and determination of islet size of mice fed with HFD or normal chow diet for 12 weeks (Insulin: red, DAPI: blue, *n* = 8). *P < 0.05; **P <0.01; ***P <0.001. All data are presented as the mean ± SEM.

Subsequently, we confirmed that NMDAR1 was expressed in the islet β cells (Figure S1B). The metabolic phenotypes were characterized to evaluate the NMDAR antagonist effect on glucose homeostasis and insulin sensitivity in mice. Following Mem treatment for 12 weeks, the fasting blood glucose level of HFD-fed mice was not statistically different from that of mice without Mem treatment (Figure 2A). Compared to control mice, HFD mice showed significantly elevated levels of insulin (Figure 2B), as well as markedly decreased glucose tolerance (IGTT, Figure 2C-D) and insulin sensitivity (IITT, Figure 2E-F). However, Mem significantly decreased blood insulin levels and improved glucose tolerance and insulin resistance induced by HFD (Figure 2B-F). Histological examination of the pancreas revealed a substantial increase in the islets area of HFD mice (Figure 2G-H). These pancreatic morphology changes were likely indicative of compensatory enlargement of β-cell mass and hypertrophy of islets in response to insulin resistance 41. Together, these results demonstrated that the NMDAR blockade improved insulin resistance and glucose homeostasis.

### Blockade of NMDAR with Mem protects against hepatic steatosis in HFD-fed mice

**Figure 3:**
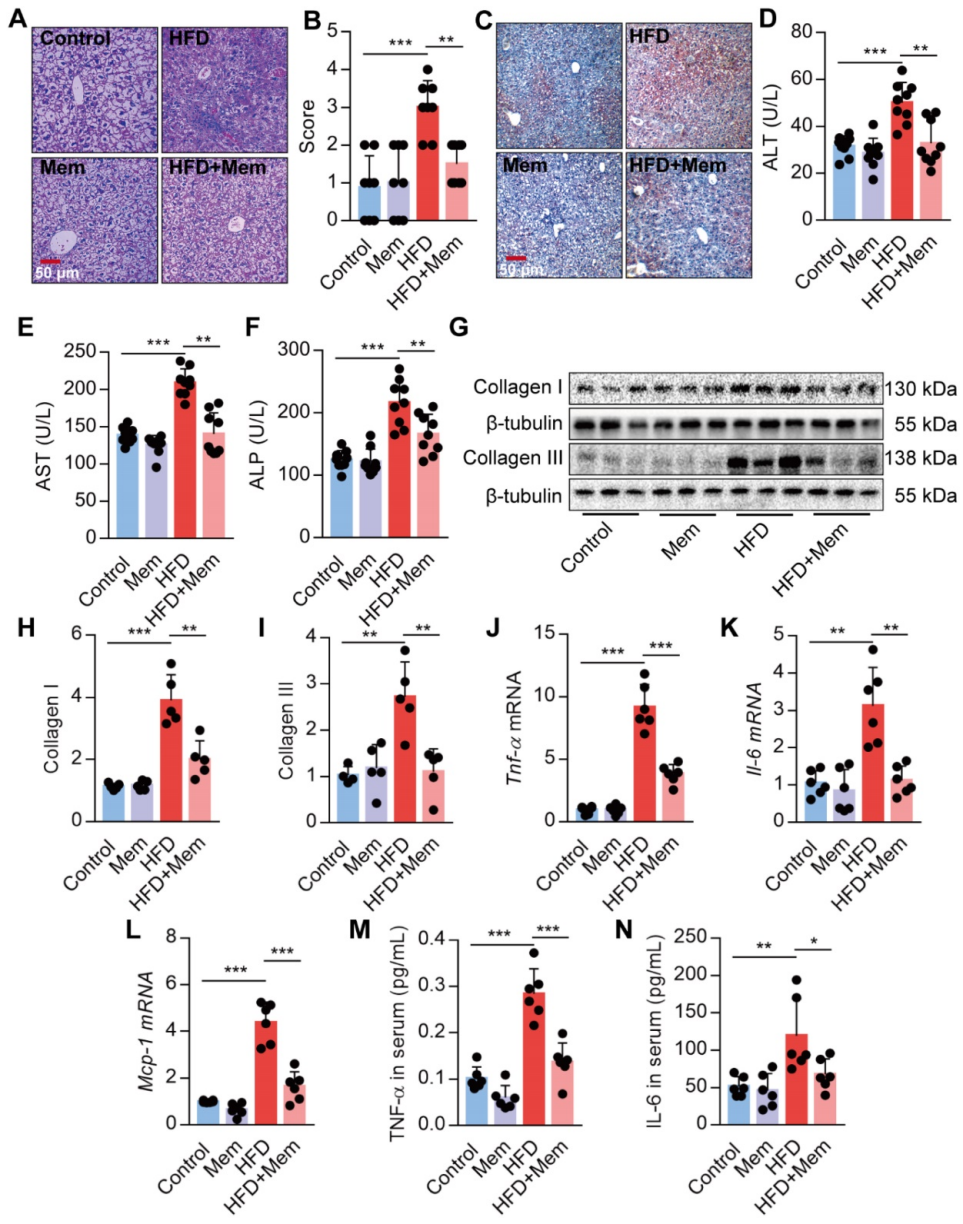
** Blockade of NMDAR by Mem prevents hepatic steatosis in mice fed with HFD.** (A-B) H&E stained histological images of liver sections from mice fed with HFD or normal chow diet for 12 weeks (Scale bars = 50 μm, *n* = 8). (C) Oil Red O staining of livers from mice fed with HFD or normal chow diet for 12 weeks (Scale bars = 50 μm). (D-F) Serum levels of ALT (D), AST (E), and ALP (F) of mice fed with HFD or normal chow diet for 12 weeks (*n* = 9). (G-I) Expression of Collagen I and Collagen III in the livers from mice fed with HFD or normal chow diet for 12 weeks by Western blotting (*n* = 5). (J-L) Expression of *Tnf-α*, *Il-6*, and *Mcp-1* mRNA in livers from mice fed with HFD or normal chow diet for 12 weeks by RT-PCR (*n* = 6). (M-N) TNF-α and IL-6 in the livers from mice fed with HFD or normal chow diet for 12 weeks by ELISA (*n* = 6). *P < 0.05; **P <0.01; ***P <0.001. All data are presented as the mean ± SEM.

Given the strong association of hepatic steatosis and hepatic insulin resistance with HFD, we performed histological comparisons between the livers from HFD mice with or without Mem treatment. The HFD-induced steatosis, lobular inflammation, and hepatocyte ballooning were significantly mitigated by MEM treatment (Figure 3A-B). Besides, Mem reduced the lipid droplets accumulated in the livers of mice challenged with HFD, as visualized by Oil Red O staining (Figure 3C). We confirmed that the NMDAR mRNAs were expressed in mouse liver tissues (Figure S1C). Mice fed with HFD exhibited significant serum AST, ALT, and ALP levels, indicating poor liver function. Mem treatment mitigated the impaired liver function (Figure 3D-F). The generation of liver fibrosis is a key feature of NAFLD 42. Mem treatement also reduced HFD-induced collagen accumulation in the liver tissues, as indicated by a significant decrease of Collagen I and Collagen III protein levels (Figure 3G-I). Moreover, the mRNA levels of inflammatory genes, including *Tnf-α*, *Il-6*, and *Mcp-1,* were also markedly reduced in Mem-treated HFD mice compared to the HFD mice (Figure 3J-L). Also, the serum concentration of TNF-α and IL-6 was significantly decreased in Mem-treated HFD mice (Figure 3M-N), although there was no significant change in MCP-1 serum level (data not shown). Taken together, these data indicated that the NMDAR blockade reduced the obesity-induced dysregulation of lipid metabolism.

### Blockade of NMDAR with Mem mitigates insulin resistance and lipid accumulation in HFD-fed mice

**Figure 4:**
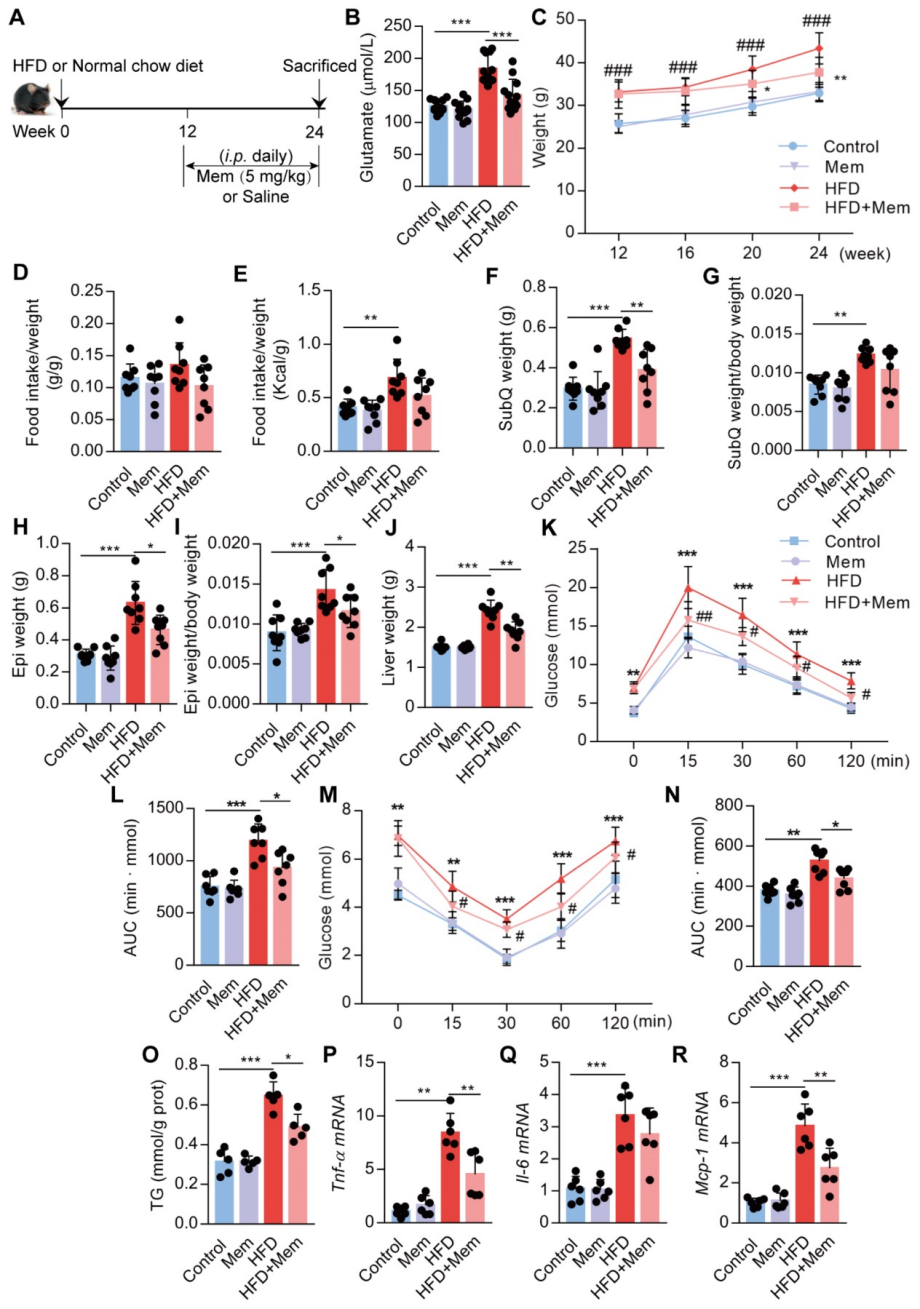
** Blockade of NMDAR by Mem mitigates insulin resistance and lipid accumulation in mice fed with HFD.** (A) C57BL/6 mice received intraperitoneal injections of Mem from week 13 to week 24 with HFD or normal chow diet. (B) Glutamate concentration in serum from mice fed with HFD for 24 weeks (*n* = 12). (C) Growth curve of mice fed with HFD or normal chow diet for 24 weeks (*n* = 12). # vs Control, ###*P* <0.001. * vs HFD, *P < 0.05; **P <0.01. (D-E) Determination of food intake of mice fed with HFD or normal chow diet for 12 weeks (*n* = 6). (F-J) Determination of SubQ, Epi, and liver weight from mice fed with HFD or normal chow diet for 24 weeks (*n* = 8). (K-L) Measurement of blood glucose during IGTT of mice fed with HFD or normal chow diet for 24 weeks (*n* = 8). Compare to the Control group, *P < 0.05; ***P <0.001. Compare to the HFD group, #*P* < 0.05; ##*P* <0.01. (M-N) Measurement of blood glucose during the IITT of mice fed with HFD or normal chow diet for 24 weeks (*n* = 7). Compare to the Control group, **P < 0.01; ***P <0.001. Compare to the HFD group, #*P* < 0.05. (O) TG content in the livers from mice fed with HFD or normal chow diet for 24 weeks was examined (*n* = 5). (P-R) Expression of *Tnf-α*, *Il-6*, and *Mcp-1* mRNA in livers was examined by RT-PCR (*n* = 6). *P < 0.05; **P <0.01; ***P <0.001. All data are presented as the mean ± SEM.

Next, we assessed whether NMDAR blockade by Mem had a therapeutic effect on obese mice fed with HFD for 12 weeks (Figure 4A). The elevated glutamate content in HFD mice was decreased (Figure 4B, Table 2) and the higher body weight was blocked (Figure 4C) by Mem administration. There were no differences in the ratio of food intake to body weight (Figure 4D). The calorie intake in HFD-fed mice was higher than standard chow diet-fed mice. However, the calorie intake of HFD-fed mice with or without Mem treatment was not statistically different (Figure 4E, Figure S1D). In parallel with the weight change, Mem-treated mice exhibited less fat mass, as indicated by a significant decrease of SubQ and Epi fat weight and liver weight (Figure 4F-J). Therapeutic Mem treatment improved systemic glucose homeostasis and insulin sensitivity (Figure 4K-N). Also, Mem-treated HFD mice had less TG content in liver tissues than HFD mice, indicating less lipid accumulation (Figure 4O). Furthermore, therapeutic Mem treatment effectively reduced the expression of *Tnf-α*, *Il-6*, and* Mcp-1* mRNA expression in the liver of HFD mice (Figure 4P-R). These results indicated that the therapeutic NMDAR blockade of NMDAR arrests arrested obesity progression by decreasing lipid accumulation, improving insulin sensitivity, and reducing inflammatory genes' expression.

### Activation of NMDAR reduces insulin sensitivity in mice fed with the normal chow diet

**Figure 5:**
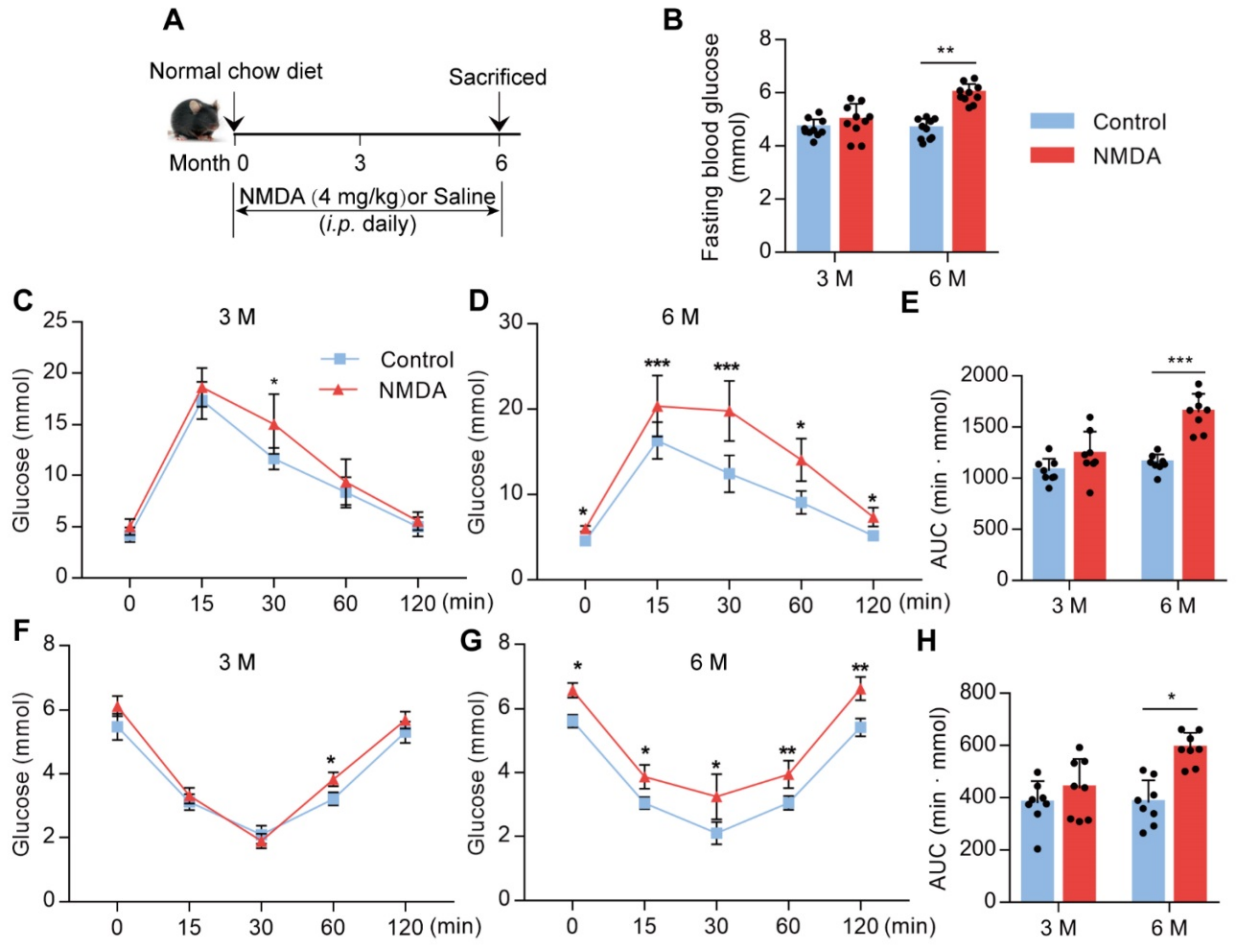
** Activation of NMDAR reduces insulin sensitivity in mice fed with a normal chow diet.** (A) C57BL/6 mice received intraperitoneal injections of NMDA from month 1 to month 6 and were fed with a normal chow diet. (B) Measurement of fasting blood glucose of mice treated with NMDA for 3 months or 6 months (*n* = 10). (C-E) Measurement of blood glucose during IGTT of mice treated with NMDA for 3 months or 6 months (*n* = 8). (F-H) Measurement of blood glucose during the IITT of mice treated with NMDA for 3 months or 6 months (*n* = 8). * vs Control, **P < 0.01; ***P <0.001. All data are presented as the mean ± SEM.

To confirm whether NMDAR activation would lead to glucose homeostasis dysregulation, we treated mice with NMDA for 3 months or 6 months (Figure 5A). There was no difference in fasting blood glucose levels between 3-month NMDA-treated and control mice. However, after 6 months of NMDA treatment, a significant increase in fasting blood glucose levels was observed (Figure 5B). Glucose tolerance and insulin tolerance tests after 3 months of NMDA treatment showed no impairment in NMDA mice. However, 6 months of NMDA treatment led to impaired glucose tolerance and insulin sensitivity, possibly because of substantial adipose tissue accumulation (Figure 5C-H). These results demonstrated that NMDAR activation resulted in insulin resistance and impaired glucose tolerance.

### Activation of NMDAR induces lipid accumulation of mice fed with a normal chow diet

**Figure 6:**
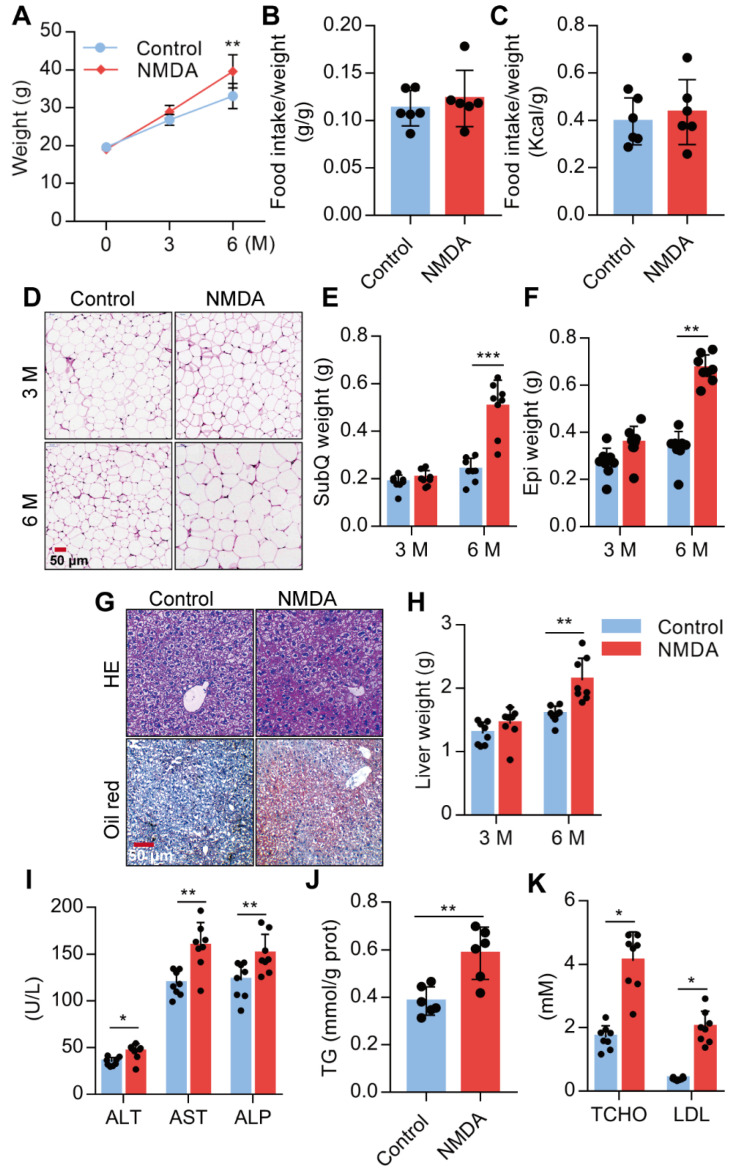
** Activation of NMDAR induces lipid accumulation in mice fed with a normal chow diet.** (A) Growth curve of mice treated with NMDA for 3 months or 6 months (*n* = 12). (B-C) Determination of food intake of mice treated with NMDA for 6 months (*n* = 6). (D) H&E staining of SubQ from mice treated with NMDA for 3 months or 6 months (Scale bars = 50 μm). (E-F) Determination of SubQ and Epi weight from mice treated with NMDA for 3 months or 6 months (*n* = 8). (G) H&E stained histological images of liver sections from mice treated with NMDA for 6 months (Scale bars = 50 μm). (H) Determination of liver weight from mice treated with NMDA for 3 months or 6 months (*n* = 8). (I) Serum levels of ALT, AST, and ALP of mice treated with NMDA for 6 months (*n* = 8). (J) TG content in livers from mice treated with NMDA for 6 months (*n* = 6). (K) Serum levels of TCHO and LDL of mice treated with NMDA for 6 months (*n* = 8). *P < 0.05, **P < 0.01, ***P < 0.001. All data are presented as the mean ± SEM.

We investigated the NMDA effect on lipid accumulation and metabolism in mice fed with a standard chow diet. We found a substantial increase in the body weight of mice treated with NMDA for 3 months and a more exaggerated increase after 6 months (Figure 6A), as NMDA did not affect the food intake of the mice (Figure 6B-C, Figure S1E). Furthermore, H&E staining of SubQ adipose tissues revealed a significantly increased adipocyte diameter in mice treated with NMDA for 6 months compared with the control group (Figure 6D). There was no difference in SubQ and Epi fat weight between control mice and mice receiving NMDA for 3 months, while NMDA treatment for 6 months significantly increased the SubQ and Epi fat weight (Figure 6E-F). Furthermore, 6 months after NMDA treatment, mice experienced significant liver enlargement and were more prone to hepatic steatosis (Figure 6G-H). The serum levels of AST, ALT, and ALP and liver TG concentration were also increased when mice were treated with NMDA for 6 months (Figures 6I-J). Blood lipid testing revealed that TCHO and LDL levels were much higher in mice after NMDA treatment for 6 months (Figure 6K). These data collectively indicated that NMDAR activation led to the dysfunction of lipid metabolism in mice fed with a standard chow diet.

### Activation of NMDAR leads to lipid accumulation through FAO impairment

**Figure 7:**
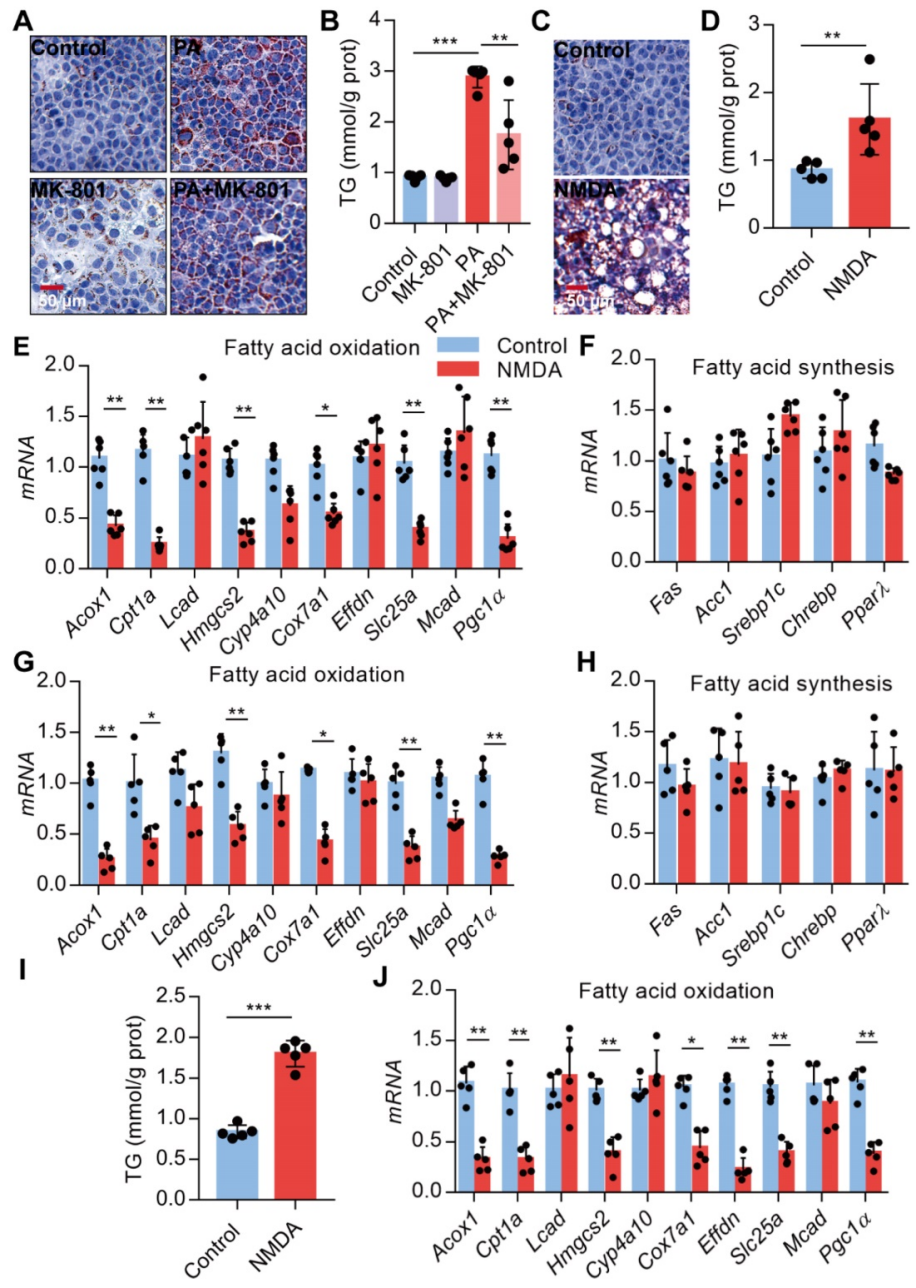
** Activation of NMDAR leads to lipid accumulation through impairing FAO.** (A) Representative images of Oil Red O staining in AML-12 hepatocytes treated with PA or/with MK-801 for 24 h (Scale bars = 50 μm). (B) TG content in AML-12 hepatocytes treated with PA or/with MK-801 for 24 h (*n* = 5). (C) Representative images of Oil Red O staining in AML-12 hepatocytes treated with NMDA for 24 h (Scale bars = 50 μm). (D) TG content in AML-12 hepatocytes treated with NMDA for 24 h was examined (*n* = 5). (E) RT-PCR analysis of genes involved in fatty acid oxidation in AML-12 hepatocytes treated with NMDA (*n* = 6). (F) RT-PCR analysis of genes involved in fatty acid synthesis in AML-12 hepatocytes treated with NMDA (*n* = 6). (G) RT-PCR analysis of genes involved in fatty acid oxidation in livers from mice treated with NMDA for 6 months (*n* = 5). (H) RT-PCR analysis of genes involved in fatty acid synthesis in livers from mice treated with NMDA for 6 months (*n* = 5). (I) TG content in HepG2 hepatocytes treated with NMDA for 24 h (*n* = 5). (J) RT-PCR analysis of genes involved in fatty acid oxidation in HepG2 hepatocytes treated with NMDA (*n* = 6). *P < 0.05, **P < 0.01, ***P < 0.001. All data are presented as the mean ± SEM.

Since NMDAR activation induces obesity phenotypes and lipid accumulation *in vivo*, we assessed NMDAR effects on lipid metabolism in hepatocytes *in vitro*. Firstly, we confirmed that the NMDAR mRNA was expressed in AML-12 cells (Figure S1F). We found that NMDAR blockade with MK-801 significantly reduced the PA-induced lipid accumulation in AML-12 cells (Figure 7A-B). Interestingly, NMDA treatment could elevate lipid droplet levels and TG content in AML-12 cells (Figure 7C-D). Next, we detected the mRNA expression of genes related to fatty acid synthesis and oxidation in AML-12 cells. RT PCR results showed that NMDA treatment significantly reduced the expression of genes involved in FAO, including *Acox1*, *Cpt1a*, *Hmgcs2*, *Cox7a1*, *Slc25a*, and* Pgc1α*(Figure 7E). While it did not affect the expression of genes related to fatty acid synthesis, including *Fas*, *Acc1*, *PPARγ*, *Sreb1c,* and *Chrebp* mRNA (Figure 7F). Consistently, NMDA treatment for 6 months caused a significant reduction of FAO-related genes in the livers of mice *in vivo* (Figure 7G-H). NMDA treatment also elevated TG content and decreased the expression of genes related to FAO in HepG2 cells (Figure 7I-J). Overall, these data indicated that NMDAR activation induced lipid accumulation by impairing FAO in hepatocytes.

### Activation of NMDAR down-regulates FAO via ERK1/2/PPARα signaling

**Figure 8:**
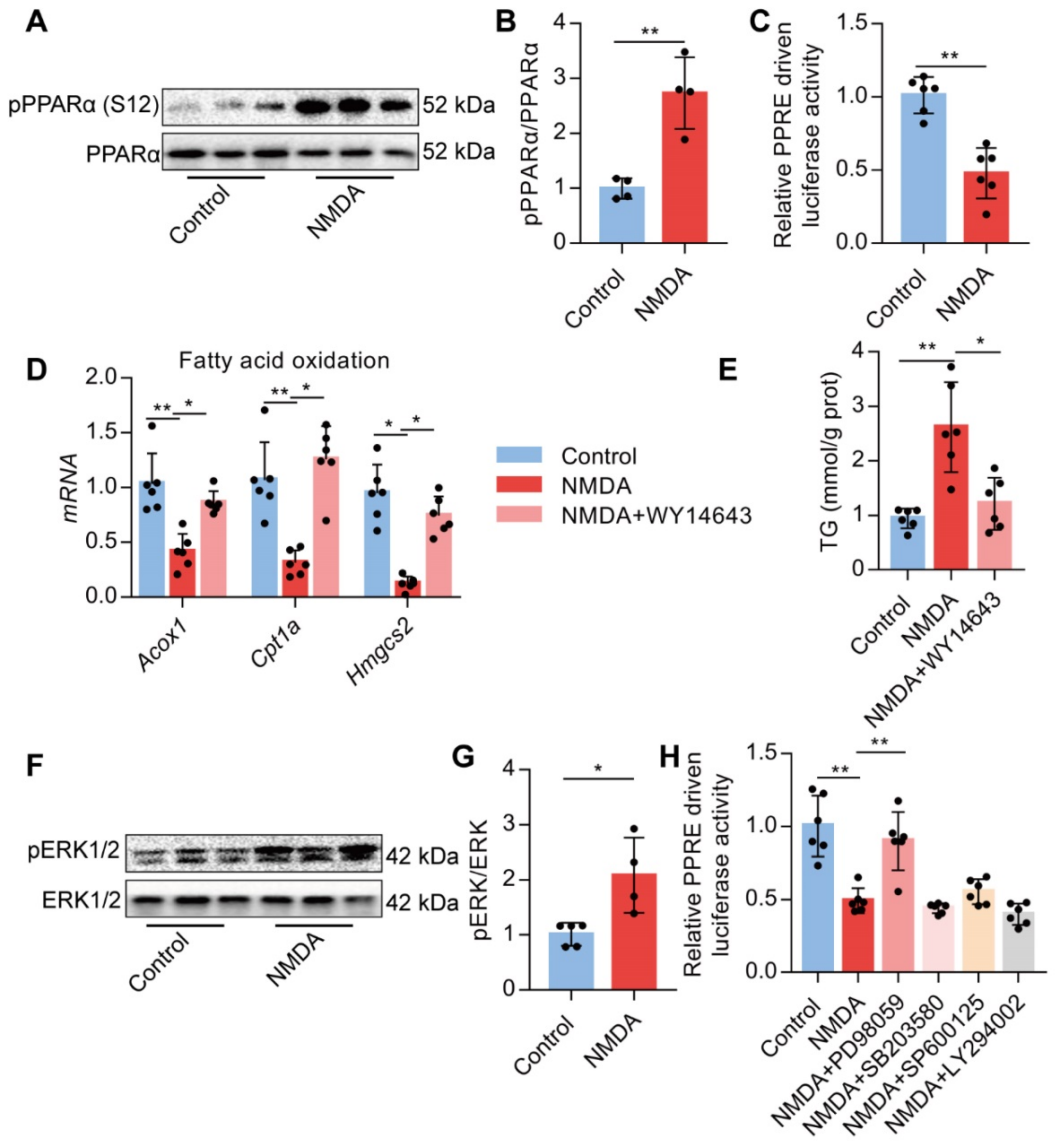
** Activation of NMDAR regulates FAO through PPARα signaling.** (A-B) pPPARα and PPARα levels in AML-12 hepatocytes treated with NMDA by Western blotting (*n* = 4). (C) Luciferase activities in AML-12 hepatocytes treated with NMDA (*n* = 5). (D) Expression of *Acox1*, *Cpt1a*, and *Hmgcs2* mRNAs in AML-12 hepatocytes treated with NMDA or WY14643 by RT-PCR (*n* = 6). (E) TG content in AML-12 hepatocytes treated with NMDA or WY14643 (*n* = 5). (F-G) Expression of pERK1/2 and ERK1/2 in AML-12 hepatocytes treated with NMDA by Western blotting (*n* = 4). (H) Luciferase activities in AML-12 hepatocytes treated with NMDA or/with PD98059, LY294002, SP600125, and SB203580 (*n* = 5). *P < 0.05, **P < 0.01. All data are presented as the mean ± SEM.

Upon activation of NMDAR by NMDA treatment, phosphorylated PPARα level at Ser-12 was significantly increased (Figure 8A-B). NMDA treatment also significantly reduced the luciferase activity of the PPARα-binding DNA element-PPRE in AML-12 cells (Figure 8C). Subsequently, we used a PPARα agonist WY14643 to investigate the role of PPARα. We found that WY14643 recovered the decreased expression of its downstream target genes Acox1, Cpt1a, and Hmgcs2 induced by NMDA (Figure 8D) and also alleviated the NMDA-induced lipid accumulation in AML-12 cells (Figure 8E). NMDA treatment also increased the phosphorylation of ERK1/2 in AML-12 cells (Figure 8F-G). Treatment with ERK1/2 inhibitor PD98059 significantly restored the luciferase activity of PPARα in hepatocytes (Figure 8H), while inhibitor for PI3K (LY294002), JNK (SP600125), and p38 (SB203580) did not significantly change the luciferase activity of PPARα (Figure 8H). These data indicated that NMDAR activation down-regulated FAO via the ERK1/2/PPARα pathway.

## Discussion

In this study, we present evidence that excessive NMDAR activation is involved in glucose and lipid homeostasis in liver tissues of mice. Blockade of NMDAR impedes HFD-induced glucose resistance and lipid accumulation and exerts a prominent therapeutic effect on HFD-induced steatosis in mice. Furthermore, overactivation of NMDAR contributes to insulin resistance, hyperlipidemia, and morphologic alterations in the liver. NMDAR blockade in hepatocytes attenuates PA-induced lipid accumulation *in vitro*. Mechanistically, NMDAR activation induces lipid accumulation through the ERK1/2/PPARα pathway in hepatocytes. From a clinical perspective, NMDAR may be a valuable therapeutic target for treating metabolic disorders.

Glutamate is the primary neurotransmitter in the mammalian CNS 20 and increased glutamate has also been demonstrated in obesity 25,26. Consistently, prolonged HFD results in an elevated level of glutamate in murine serum, indicating that the excessive increase of glutamate plays a key role in developing metabolic disorders. Recently, the presence of functional glutamate receptors (GluRs) has been demonstrated in non-neuronal tissues and cells, including the kidney, lung, and urogenital tract 43,44. GluRs are categorized into two major classes of metabotropic (mGluRs) and ionotropic (iGluRs) receptors 45. However, little is known about the expression and role of the GluRs in the liver. The presence of both GluRs, including iGluR expression, such as NMDAR1 and KA2, has been reported in the liver 46,47. mGluR5 in rat hepatocytes could be activated by glutamate in the portal blood and contributed to liver damage under adverse conditions 47. Here, we found that the liver and hepatocytes expressed NMDA-type glutamate receptors, including NMDAR1 and NMDAR2A-2D. These findings suggested that the functional expression of NMDAR in hepatocytes play crucial roles in regulating lipid metabolism. Further studies are required to decipher the molecular details of glutamate signaling through its different NMDA-type receptors in the liver.

The treatment efficacy of an NMDAR inhibitor, Mem (5 mg/kg weight), in obese mice identifies NMDAR as a potential therapeutic target for lipid accumulation and insulin resistance in obese patients. It has been reported that Mem (20 mg/kg) treatment for 18 days caused increased weight loss in HFD-induced obese mice 48. Another study has shown that preference for HFD could be decreased by low-dosage of NMDA receptor antagonists, ketamine, ifenprodil, or MK-801 49. Mem could also reduce binge-like eating of a sucrose diet 50. These results are presumably attributed to the decreased food intake through an NMDAR-dependent neural effect. Moreover, HFD alters several aspects of glutamate, dopamine, and opioid signaling within the dorsal striatum. These alterations known to play a role in food motivation/consumption and habitual behaviors are highly relevant for clinical obesity and its treatment 51. However, in our study, we found that the food calorie intake of HFD-induced mice was not statistically different from that treated with or without Mem, suggesting that Mem decreased HFD induced body weight through an NMDAR-dependent non-neural effect. Conversely, chronic NMDA injections (25mg/Kg) resulted in increased body weight and reduced brain PLA2 activity 52. This finding is consistent with our results showing that exogenous NMDA treatment for 6 months caused an extensive lipid accumulation and decreased insulin sensitivity. These data suggested a deleterious effect of NMDAR activation on metabolic regulation. Therefore, excessive activation of NMDAR as appeared to be an underlying mechanism of metabolic-related disorders.

Our previous studies have demonstrated that NMDAR mRNA was expressed in the islets and the β-cells 24. We also confirmed the expression of NMDAR1 protein in the β-cells by double-labeling immunofluorescence. Blockade of NMDAR increased insulin secretion and improved β cell function in diabetic mice 24,36. In the present study, we found that treatment with NMDAR blocker prevents insulin resistance and promotes glucose tolerance induced by HFD. It has been reported that gut infusion of ghrelin inhibited increased hepatic glucose production and expression of gluconeogenic enzymes and decreased insulin signaling in the rat liver. Co-infusion with the NMDAR inhibitor negated gut ghrelin effects within the dorsal vagal complex 53. However, further studies are required to assess whether NMDA-induced insulin resistance is dependent on the activation of NMDAR in the liver.

The imbalance between fatty acid synthesis and FAO has been shown to contribute to hepatic steatosis in obese mice 54. FAO involves the degradation of fatty acids by sequential removal of two‑carbon units from the acyl chain to produce acetyl-CoA 16,18. It has been demonstrated that inadequate hepatic FAO leads to hepatic lipid disorders and massive steatosis 55. Here, we explored the role of NMDAR in FAO and showed that NMDAR activation regulated lipid homeostasis by altering FAO in hepatocytes in vitro. Accumulating evidence has shown that hepatic FAO is impaired in human liver diseases 56. We found that NMDAR activation developed massive steatosis and insulin resistance in mice due to impaired FAO.

PPARα is the major controlling factor for FAO and energy generation under a nutrient-deprived state 55,57. FAO was significantly reduced in livers of mice lacking PPARα 58,59. Hepatocyte PPARα-specific knockout mice exhibited severe HFD-induced hepatic steatosis 60,61. PPARα has also been shown to regulate cellular metabolism and inflammatory response through FAO 62. The study further demonstrated that PPARα in the hippocampus controls calcium influx and the expression of several genes coding hippocampal proteins involved in synaptic plasticity regulation 62. Furthermore, PPAR-α is involved in the expression of NMDAR2A and NMDAR2B genes 63. Pharmacological NMDAR antagonists lack specificity and have other CNS-related effects. However, we confirmed a direct role of NMDAR activation in PPARα activity *in vitro*, suggesting that at least part of the NMDAR antagonist's effects may be ascribed to the peripheral NMDAR blockade. We further demonstrated that inhibition of PPARα by its agonists such as WY14643 decreased TG levels induced by activation of NMDAR. Phosphorylation of PPARα is an efficient mechanism whereby its activity can be modulated 64,65. We confirmed that alterations in hepatic PPARα activities induced by NMDA were associated with PPARα phosphorylation status at the Ser-12 phosphorylated site. Both Ser-21 and Ser-12 were previously demonstrated to be the ERK-mediated phosphorylation sites on PPARα in cardiac myocytes, and inhibition of their phosphorylation by ERK inhibitor PD98059 caused increased transcription of CPT1 66. It has also been reported that aldose reductase plays an important role in the regulation of hepatic PPARα phosphorylation at Ser-21 through ERK1/2 signaling 65. Finally, we demonstrated that PD98059 significantly restored the activity of PPARα, implying ERK1/2 to be upstream of PPARα in hepatocytes treated with NMDA. Together these data indicated that activation of NMDAR could greatly decrease PPARα activity.

In conclusion, the excessive activation of NMDAR may represent the underlying pathogenesis of metabolic disorders induced by HFD in mice. Overactivation of NMDAR impedes FAO by reducing PPARα activity in hepatocytes. These findings reveal a new mechanism underlying the NMDAR' role in glucose and lipid metabolism and identify a promising target for treating hepatic steatosis and insulin resistance by blockade of NMDAR.

## Supplementary Material

Supplementary figure and table.

## Figures and Tables

**Table 1:** Glutamate concentration in serum from mice fed with HFD for 12 weeks (n=12)

Group	Glutamate (μmol/L)
Control	106.11±22.95
Mem	115.71±17.27
HFD	149.20±30.19***
HFD+Mem	122.72±16.80^##^

Compare to the Control group, ***P <0.001. Compare to the HFD group, ##*P* <0.01.

**Table 2:** Glutamate concentration in serum from mice fed with HFD for 24 weeks (n=12)

Group	Glutamate (μmol/L)
Control	125.69±9.78
Mem	119.97±13.49
HFD	183.47±22.26***
HFD+Mem	142.82±24.66^###^

Compare to the Control group, ***P <0.001. Compare to the HFD group, ###*P* <0.001.

## Abbreviations

ALP: alkaline phosphatase
ALT: alanine aminotransferase
AST: aspartate aminotransferase
AUC: area under the curve
CNS: central nervous system
Epi: epididymal fat pad
ERK1/2: extracellular signal-regulated kinase ½
FAO: fatty acid oxidation
HFD: high-fat diet
IGTT: intraperitoneal glucose tolerance test
IITT: intraperitoneal insulin tolerance test
Il-6: interleukin-6
LDL: low-density lipoprotein
MCP-1: monocyte chemotactic protein 1
Mem: memantine
NAFLD: non-alcoholic fatty liver disease
NMDA: N-methyl-D-aspartate
NMDAR: N-methyl-D-aspartate receptor
PA: palmitic acid
PPARα: peroxisome proliferators-activated receptor α
PPRE: peroxisome proliferator response elements
SubQ: subcutaneous fat
T2D: type 2 diabetes
TCHO: total cholesterol
TG: triglyceride
TNF-α: tumor necrosis factor-α
