# Supplementary material for: Activation of N-methyl-D-aspartate receptor regulates insulin sensitivity and lipid metabolism
Source: Theranostics. 2021 Jan 1;11(5):2247–62. doi: 10.7150/thno.51666 (PMC7797674; doi:10.7150/thno.51666)
Supplement: Supplementary file 1 — Supplementary figure and table. [file thnov11p2247s1.pdf]

Supplementary Figure

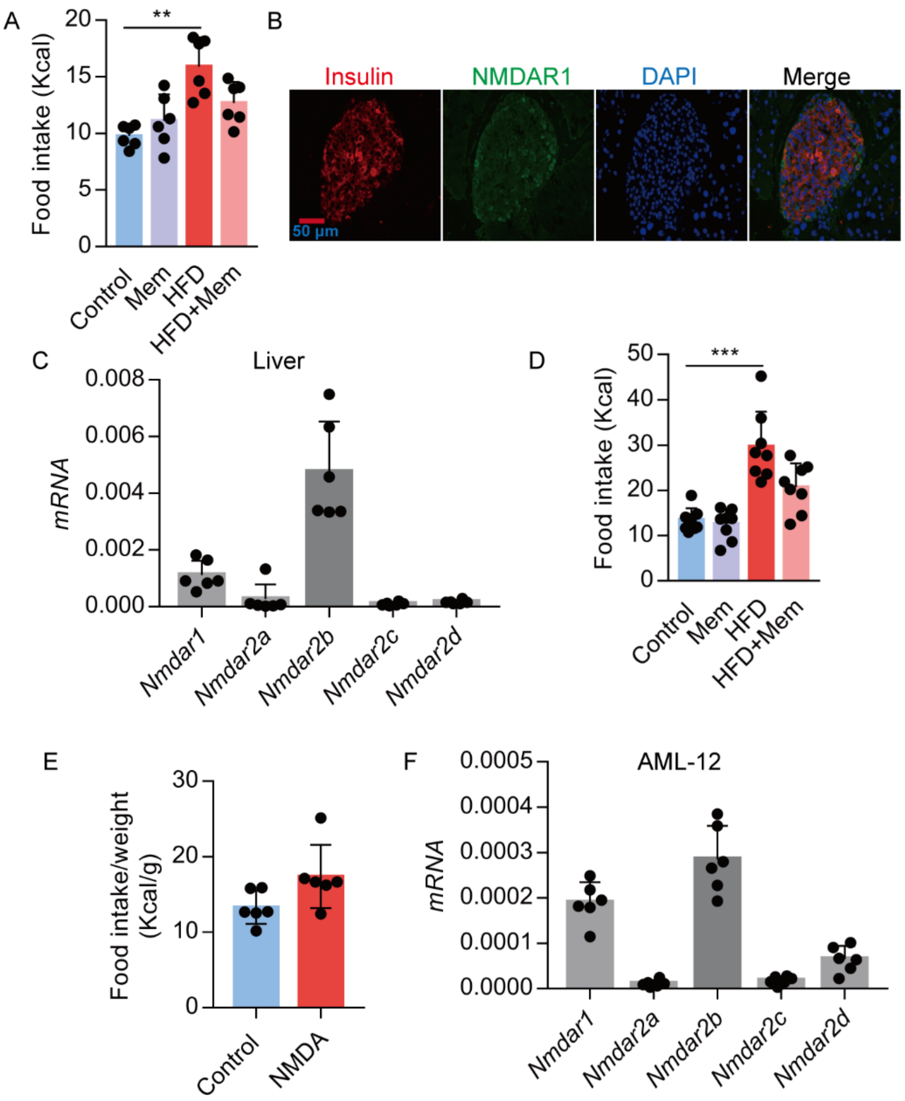

Sup Figure 1. (A) Determination of food intake of mice fed with HFD or normal chow diet for 12 weeks (n = 6). (B) Representative immunofluorescence staining images of pancreases in control mice (Insulin: red, NMDAR1: Green, DAPI: blue, n = 4). (C) RT-PCR analysis of NMDAR genes in liver tissue of mice (n = 6). (D) Determination of food intake of mice fed with HFD or normal chow diet for 24 weeks (n = 8). (E) Determination of food intake of mice treated with NMDA for 6 months (n = 6). (F) RT-PCR analysis of NMDAR genes in AML-12 hepatocytes (n = 6). **$P < 0.01$, ***$P < 0.001$. All data are presented as the mean ± SEM.

Supplementary Table

Table 1. Sequences of specific primers used in this study

Gene	Species	Forward primer (5′–3′)	Reverse primer (5′–3′)
Acox1	mouse	CCTGATTCAGCAAGGTAGGG	TCGCAGACCCTGAAGAAATC
Cpt1a	mouse	AGTGGCCTCACAGACTCCAG	GCCCATGTTGTACAGCTTCC
Hmgcs2	mouse	ATACCACCAACGCCTGTTATG	CAATGTCACCACAGACCACCA
Cyp4a10	mouse	AAGGGTCAAACACCTCTGGA	GATGGACGCTCTTTACCCAA
Pparγ	mouse	GCTGTTATGGGTGAAACTCT	TGGCATCTCTGTGTCAACCA
Cox7a1	mouse	GTCTCCCAGGCTCTGGTCCG	CTGTACAGGACGTTGTCCATTC
Pgc1α	mouse	TCCTCCTCATAAAGCCAACC	GCCTTGGGTACCAGAACACT
Mcad	mouse	CCAGAGAGGAGATTATCCCCG	TACACCCATACGCCAACTCTT
Srebp1c	mouse	GTTACTCGAGCCTGCCTTCAGG	CAAGCTTTGGACCTGGGTGTG
Acc1	mouse	GGACAGACTGATCGCAGAGAAAG	TGGAGAGCCCCACACACA
Fas	mouse	GCTGCGGAAACTTCAGGAAAT	AGAGACGTGTCACTCCTGGACTT
Lcad	mouse	GCATCAACATCGCAGAGAAA	TCGCAATATAGGGCATGACA
Etfdh	mouse	GTCTTGATCCAGCTGCCTTC	ACCTGGAAGAATTGGCACAG

Chrebp	mouse	CCTCACTTCACTGTGCCTCA	ACAGGGGTTGTTGTCTCTGG
Mcp-1	mouse	GTCCCTGTCATGCTTCTGG	GCGTTAACTGCATCTGGCT
Il-6	mouse	CTGGGGATGTCTGTAGCTCA	CTGTGAAGTCTCCTCTCCGG
Tnf-α	mouse	AGCCCCCAGTCTGTATCCTT	CTCCCTTTGCAGAACTCAGG
β-actin	mouse	TTCCAGCCTTCCTTCTTG	GGAGCCAGAGCAGTAATC
Nmdar1	mouse	ACTCCCAACGACCACTTCAC	GTAGACGCGCATCATCTCAA
Nmdar2a	mouse	AGACCTTAGCAGGCCCTCTC	CTCTTGCTGTCCTCCAGACC
Nmdar2b	mouse	CCGCAGCACTATTGAGAACA	ATCCATGTGTAGCCGTAGCC
Nmdar2c	mouse	ATCGGGGTCAACAATACCAA	CACAGCAGAACCTCCACTGA
Nmdar2d	mouse	TAGTGTCAGTGCGCAGATCC	TCCTGGCAGAAGAAGTGGTT
Lcad	human	AAGCGAAACGTTTGGACTCC	CCCACATGTATCCCCAACCT
Etfdh	human	GCTCTTGGTCTTGTGGTTGG	AGCCACCTTCATTGAGAGCT
Acox1	human	GGGACCCATAAGCCTTTGCC	CTTGTTACTACGCGGTTTCACG
Cpt1a	human	ATCAATCGGACTCTGGAAACGG	ATCTTGGTGGCACGACTCATCT
Hmgcs2	human	TCTGTCCCCTTGCAATTCCA	TAGAACAGGGAAGTGGGCTG
Cox7a1	human	GTACCGAGTGACAATGACGC	GGCCAGCGTTTATTGACACT
Pgc1α	human	CACCAGCCAACACTCAGCTAAG	AGGGTCATCGTTTGTGGTCAG
Mcad	human	TGTGGAAGCAGATACCCCAG	ACCAGCTCCGTCACCAATTA
Gapdh	human	CCAAGGAGTAAGACCCCTGG	AGGGGAGATTCAGTGTGGTG
